# Lipoteichoic Acid (LTA) and Lipopolysaccharides (LPS) from Periodontal Pathogenic Bacteria Facilitate Oncogenic Herpesvirus Infection within Primary Oral Cells

**DOI:** 10.1371/journal.pone.0101326

**Published:** 2014-06-27

**Authors:** Lu Dai, Michael R. DeFee, Yueyu Cao, Jiling Wen, Xiaofei Wen, Mairi C. Noverr, Zhiqiang Qin

**Affiliations:** 1 Research Center for Translational Medicine and Key Laboratory of Arrhythmias. East Hospital, Tongji University School of Medicine, Shanghai, China; 2 Department of Microbiology/Immunology/Parasitology, Louisiana State University Health Sciences Center, Louisiana Cancer Research Center, New Orleans, Louisiana, United States of America; 3 Department of Medicine, Louisiana State University Health Sciences Center, Louisiana Cancer Research Center, New Orleans, Louisiana, United States of America; 4 Department of Oral Health Sciences, Medical University of South Carolina, Charleston, South Carolina, United States of America; 5 Department of Oral and Craniofacial Biology, Louisiana State University Health Sciences Center, Louisiana Cancer Research Center, New Orleans, Louisiana, United States of America; 6 Department of Urology, East Hospital, Tongji University School of Medicine, Shanghai, China; Lisbon University, Portugal

## Abstract

Kaposi’s sarcoma (KS) remains the most common tumor arising in patients with HIV/AIDS, and involvement of the oral cavity represents one of the most common clinical manifestations of this tumor. HIV infection incurs an increased risk for periodontal diseases and oral carriage of a variety of bacteria. Whether interactions involving pathogenic bacteria and oncogenic viruses in the local environment facilitate replication or maintenance of these viruses in the oral cavity remains unknown. In the current study, our data indicate that pretreatment of primary human oral fibroblasts with two prototypical pathogen-associated molecular patterns (PAMPs) produced by oral pathogenic bacteria–lipoteichoic acid (LTA) and lipopolysaccharide (LPS), increase KSHV entry and subsequent viral latent gene expression during *de novo* infection. Further experiments demonstrate that the underlying mechanisms induced by LTA and/or LPS include upregulation of cellular receptor, increasing production of reactive oxygen species (ROS), and activating intracellular signaling pathways such as MAPK and NF-κB, and all of which are closely associated with KSHV entry or gene expression within oral cells. Based on these findings, we hope to provide the framework of developing novel targeted approaches for treatment and prevention of oral KSHV infection and KS development in high-risk HIV-positive patients.

## Introduction

Infection with the Kaposi’s sarcoma-associated herpesvirus (KSHV) and subsequent development of its principal clinical consequence–Kaposi’s sarcoma (KS) [1] –occur with greater frequency following HIV infection or organ transplantation [2], [3]. Despite the reduced incidence of KS after employing highly active antiretroviral therapy (HAART) for HIV infection [4], [5], KS remains the most common AIDS-associated tumor and a leading cause of morbidity and mortality in this setting [2]. Existing clinical data suggest that KSHV dissemination within and from the oral cavity are critical factors for KSHV infection and oral KS progression in HIV-infected patients [6]–[10]. Person-to-person transmission of KSHV is thought to occur primarily through exchange of oropharyngeal secretions [6], [7], and epidemiologic data indicate that sexual practices involving contact with the oral cavity promote KSHV transmission [8]. Furthermore, HAART does not reduce KSHV replication within the oropharynx [6], [8] or KSHV transmission [10]. These data are congruous with data collected from patients in North America (including the U.S.) suggesting that the prevalence of intraoral KS has not declined significantly in the HAART era [11], [12].

Periodontitis is characterized by chronic inflammation associated with oral bacteria, resulting in destruction of periodontal ligaments and supporting bone of the tooth [13]. Several studies indicate a significantly higher prevalence of severe oral inflammation and periodontal disease for HIV-positive patients [14], [15]. Pathogenesis of periodontitis and other oral inflammation is dependent on the local microbiome within the gingival sulcus, and studies of the microbiota indicate that many of the same bacteria contributing to periodontitis in otherwise healthy persons also likely contribute to periodontitis for HIV-positive patients, including *Porphyromonas gingivalis* [16], [17]. In contrast, some pathogens associated with periodontitis have been found more commonly in the setting of HIV infection, including *Staphylococcus aureus* [17]. Moreover, published literatures have indicated increased methicillin-resistant *S. aureus* (MRSA) colonization and incidence of severe invasive infection in HIV-infected population especially HIV-infected children [18]–[20].

Oral KS lesions display higher KSHV viral loads and may portend more ominous prognoses relative to KS in other anatomic locations [21], [22], but whether this is due to interactions between KSHV and oral pathogenic bacteria is unknown. Published data have reported that the interactions between periodontal bacteria and viruses facilitate periodontal disease, and some periodontal bacteria promote viral infection and replication [23]–[25]. Interestingly, herpesviruses, including Epstein-Barr virus and cytomegalovirus, occur at high copy counts in aggressive periodontitis, potentially through impairing local host defenses and thus increasing the aggressiveness of resident periodontopathic bacteria [26]. Pathogen-associated molecular patterns (PAMPs) produced by multiple bacterial species are recognized by pathogen recognition receptors (PRRs) and induce host cell innate immune responses [27]. Lipoteichoic acid (LTA) and Lipopolysaccharides (LPS), represent two major PAMPs molecules produced by Gram-positive and Gram-negative bacterial species, respectively. Both LTA and LPS can interact with many host factors or regulate intracellular signaling pathways to induce host immune response, therefore contributing to bacterial pathogenesis [28], [29]. In addition, LTA and LPS represent important immunogenic components in those most common bacteria associated with dental diseases including periodontitis [30], [31].

We recently reported that KSHV successfully established latent infection in primary human gingival fibroblasts (HGF) or periodontal ligament fibroblasts (PDLF) *in vitro*, and virus *de novo* infection induced a tumor-associated fibroblast (TAF)-like phenotype within these cells [32]. Other published data also demonstrated that fibroblasts represented an imporant component within KS lesions and supported *de novo* KSHV infection [33], [34]. KSHV-infected endothelial cells represent the predominant component of KS lesions, however, to our knowledge, primary endothelial cells or endothelial cell lines derived specifically from the oral cavity are not commerically available now. Therefore, in the current study we have continuously used these two primary oral fibroblasts to determine whether *S. aureus*-derived LTA and/or *P. gingivalis*-derived LPS impact KSHV infection (including viral entry and consequently viral gene expression) and the underlying complex mechanisms.

## Results

### LTA and LPS from periodontal pathogenic bacteria facilitate KSHV viral entry and latent gene expression during de novo infection of primary oral fibroblasts

**Figure 1:**
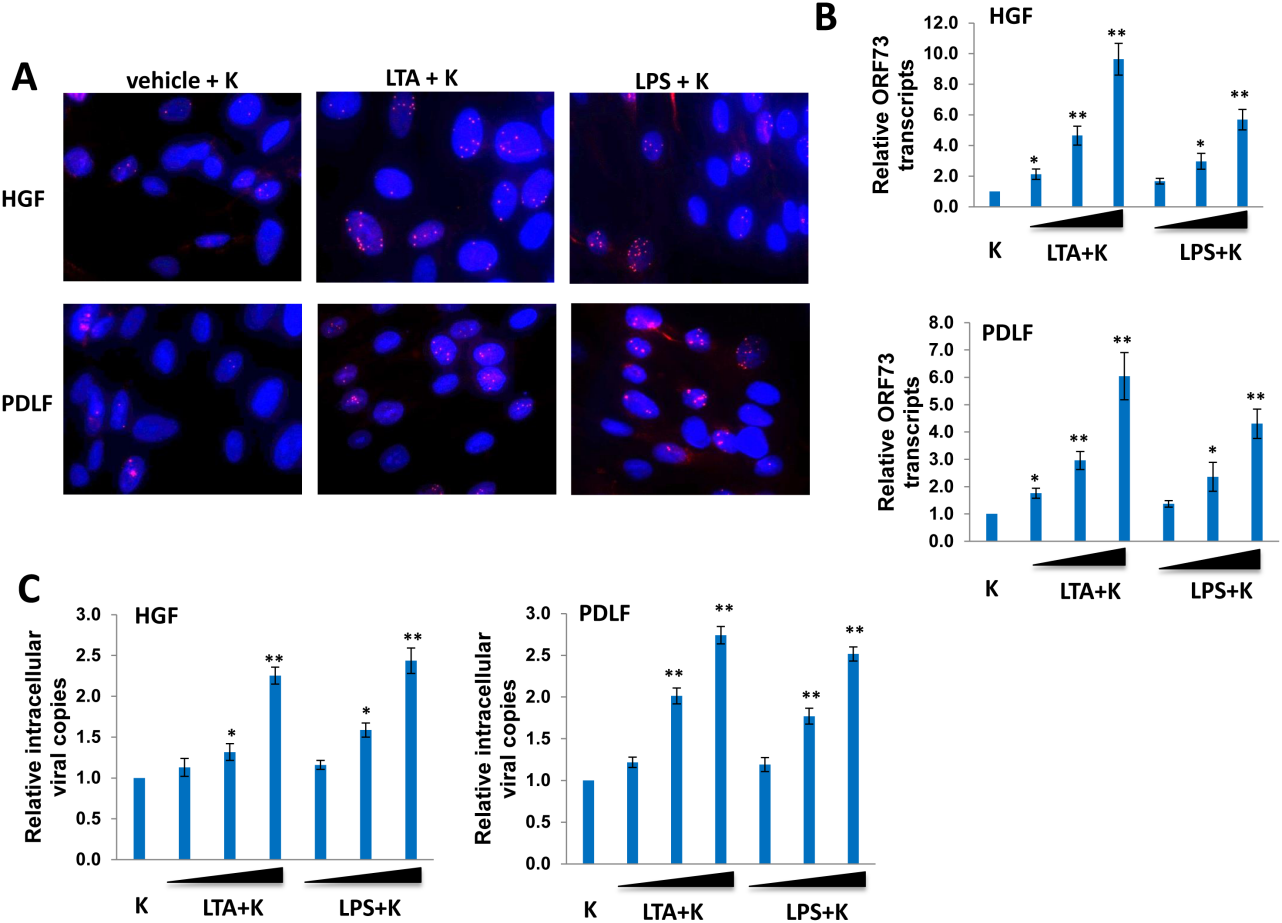
LTA and LPS from periodontal pathogenic bacteria increase KSHV entry and latent gene expression during *de novo* infection of primary oral cells. (A) HGF and PDLF cells were pre-treated with 5 µg/mL of purified LTA from *S. aureus* or LPS from *P. gingivalis* for 24 h, then infected with purified KSHV (MOI∼3). IFA to identify LANA expression were performed 24 h p.i. using an anti-LANA monoclonal antibody and a secondary antibody conjugated to Texas Red, along with DAPI for nuclear colocalization (blue). (B–C) HGF and PDLF were pre-treated with LTA or LPS (2.5, 5, 10 µg/mL, respectively) for 24 h, then infected with purified KSHV as (A). qRT-PCR was used to quantify *ORF73* (*Lana*) transcripts at 24 h p.i (B). qPCR was used to quantify intracellular KSHV DNA contents 2 h after incubation of cells with the virions (C). Error bars represent the standard errors of the means for 3 independent experiments. * = p<0.05, ** = p<0.01.

We first sought to determine whether LTA and LPS from commonly periodontal pathogenic bacterial species including *S. aureus* and *P. gingivalis*, respectively, affected KSHV infection of primary human oral cells. Therefore, we pre-treated HGF and PDLF with 5 µg/mL of purified LTA from *S. aureus* or LPS from *P. gingivalis* for 24 h, then followed by incubation with purified KSHV virions (MOI∼3). Immunofluorescence assays (IFA) results revealed that either LTA or LPS pretreatment apparently increased intranuclear expression of the KSHV-encoded latency-associated nuclear antigen (LANA) (Fig. 1A), which represented the major marker for establishment of viral latency within HGF and PDLF [32]. Next, qRT-PCR data confirmed that LTA or LPS pretreatment significantly increased *ORF73* (*Lana*) transcripts within oral fibroblasts, respectively, in a dose-dependent manner (Fig. 1B). Subsequently, we tried to determine whether periodontal bacterial LTA or LPS pretreatment also have impacts on early infection stage especially virus entry into oral cells. Our qPCR data indicated that LTA or LPS pretreatment significantly increased KSHV entry (represented by internalized intracellular viral copies) within 2 hours of KSHV incubation with HGF and PDLF, as well as in a dose-dependent manner (Fig. 1C). In addition, MTT assays indicated that the doses of LTA and LPS used in the current study did not reduced visible cell viability for oral fibroblast (Fig. S1), implying that the above observations by LTA and LPS are not due to altering cell viability.

### Periodontal bacterial LTA and LPS promote KSHV entry into oral fibroblasts potentially through upregulation of cellular receptor

**Figure 2:**
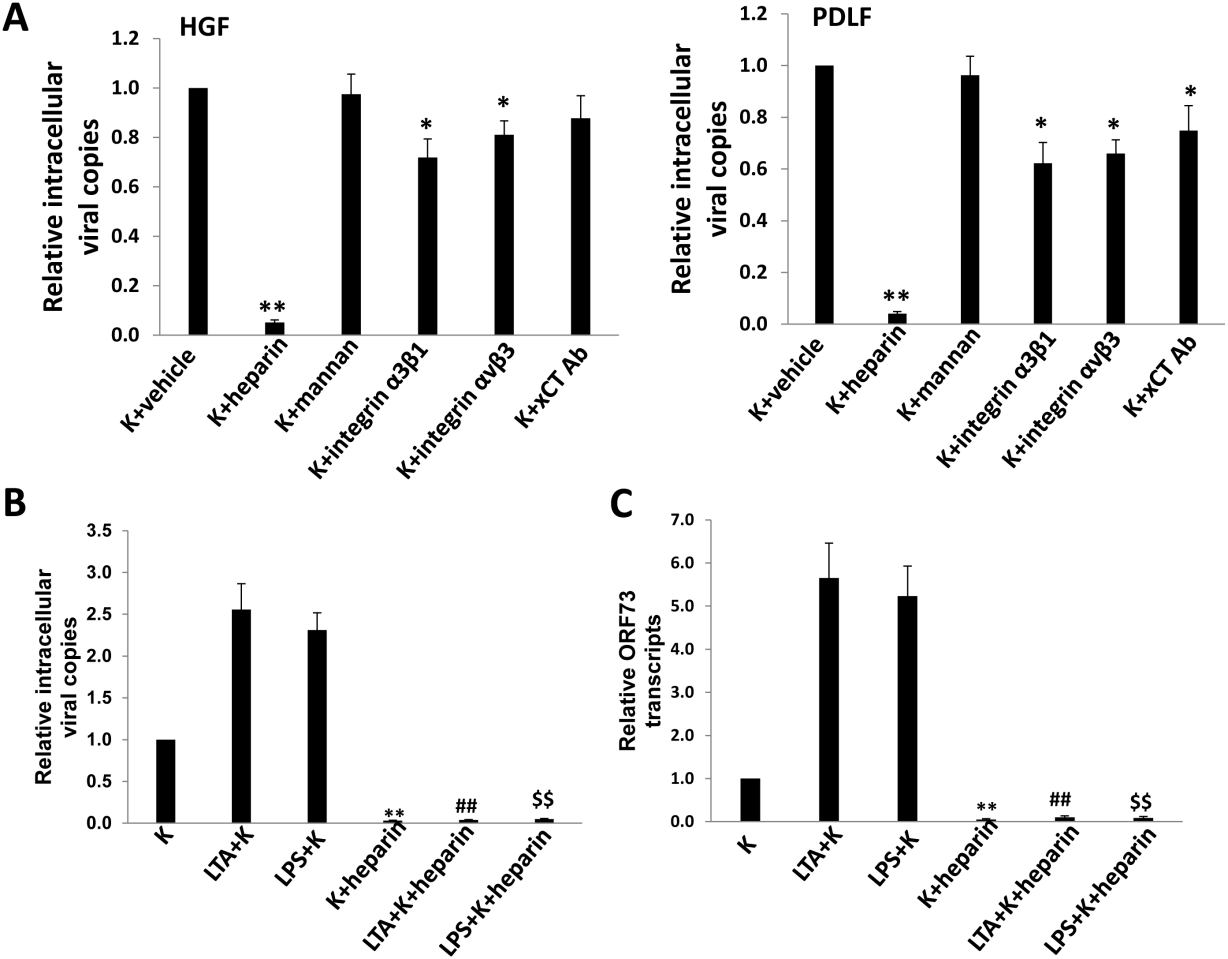
Heparan sulfate is the major cellular receptor responsible for KSHV entry into oral fibroblasts. (A) HGF and PDLF were first treated with 0.4 mg/mL mannan (the inhibitor of DC-SIGN) or 20 µg/mL xCT Ab, or purified virions were first treated with 0.5 mg/mL heparin (the competitor for heparan sulfate) or 15 µg/mL soluble integrin α3β1 and αvβ3 for 1 h at 4°C, then the cells were infected with purified virions (MOI∼3) for 2 h at 37°C. After that, cells were trypsinized and washed to remove extracellular KSHV virions. Total cellular DNA was prepared and the internalized viral copies were measured by qPCR as described in Methods. Error bars represent the standard errors of the means for 3 independent experiments. * = p<0.05, ** = p<0.01. (B–C) PDLF were incubated with 10 µg/mL LTA or LPS for 24 h, and purified virions (MOI∼3) were incubated with or without 0.5 mg/mL heparin for 1 h at 4°C. Cells were subsequently infected for 2 h at 37°C, then DNA (2 h p.i.) and RNA (24 h p.i.) were isolated for quantification of intracellular viral copies or *ORF73* (*Lana*) transcripts using qPCR (B) or qRT-PCR (C), respectively. **/##/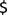 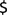 = p<0.01 relative to K (**), LTA+K (##), and LPS+K (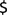 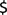).

**Figure 3:**
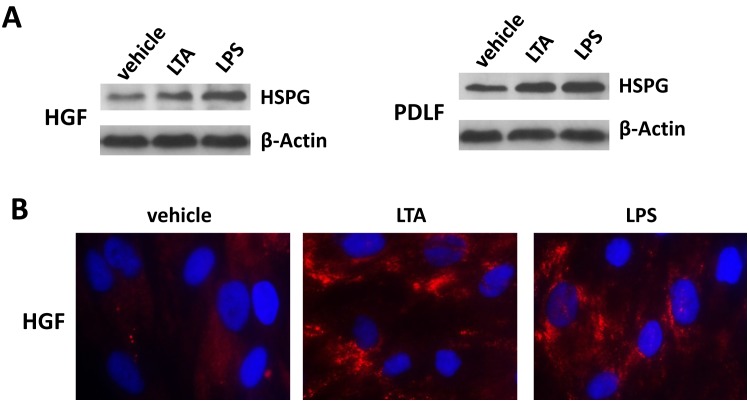
LTA and LPS from periodontal pathogenic bacteria increase heparan sulfate proteoglycans (HSPG) expression in HGF and PDLF. (A) HGF and PDLF cells were pre-treated with 10 µg/mL of LTA from *S. aureus* or LPS from *P. gingivalis* for 24 h, then proteins expression was detected by immunoblots. (B) HGF were treated as (A), and total HSPG expression was detected by IFA as described in Methods.

One of possible mechanisms underlying for increasing KSHV entry is through upregulation of its cellular receptors, and some of which have been identified within a variety of host cells, including Heparan sulfate (HS), DC-SIGN, Integrin α3β1, αvβ3 and xCT [35]–[39]. However, it remains unclear which cellular receptors are responsible for KSHV entry into oral cells including fibroblast. Therefore, we first tested the effects of blocking virus entry by using various strategies targeting respective viral receptors as described in Methods. The results concluded as follows for HGF and PDLF: 1) Heparin, the competitor of HS, had the maximal effect on blocking viral entry (>90%); 2) soluble Integrin α3β1, αvβ3 or xCT antibody only partially reduced viral entry (∼20%–30%); 3) Mannan, the DC-SIGN inhibitor, almost had no effects on viral entry (Fig. 2A). Furthermore, pre-incubation of purified KSHV virions with heparin significantly blocked LTA/LPS-induced virus entry and consequently viral gene expression in PDLF (Fig. 2B–C). We also observed similar results in HGF (Fig. S2). Taken together, our data indicated that HS is the major cell-surface receptor on oral fibroblasts for KSHV entry. HS is a linear polysaccharide, which occurs as a proteoglycan (HSPG) on cell surface [40]. Therefore, we next tested whether oral bacterial LTA and LPS can regulate HSPG expression in oral cells using a specific HSPG antibody. As shown in Fig. 3, both immunoblots and IFA confirmed the upregulation of HSPG by LTA or LPS in oral fibroblasts. In addition, we detected the expression of other cellular receptors (Integrin α3β1, αvβ3, xCT and DC-SIGN) on cell-surface of oral fibroblasts with or without LTA or LPS treatment by flow cytometry (Fig. S3). The results confirmed that their expressions were not affected by either LTA or LPS treatment, and DC-SIGN was found almost not expressed on cell-surface. Actually, we also tried to test HSPG expression by flow cytometry but unfortunately we found that this HSPG antibody cannot be used to detect its expression on cell-surface by flow cytometry (data not shown). We sought to try alternative HSPG antibody which has been reported to detect its cell-surface expression by flow cytometry [41], [42], but found that it has been stopped supplied by Seikagaku Corp (Tokyo, Japan).

### Periodontal bacterial LTA and LPS increase KSHV entry and viral gene expression through inducing reactive oxygen species (ROS) production

**Figure 4:**
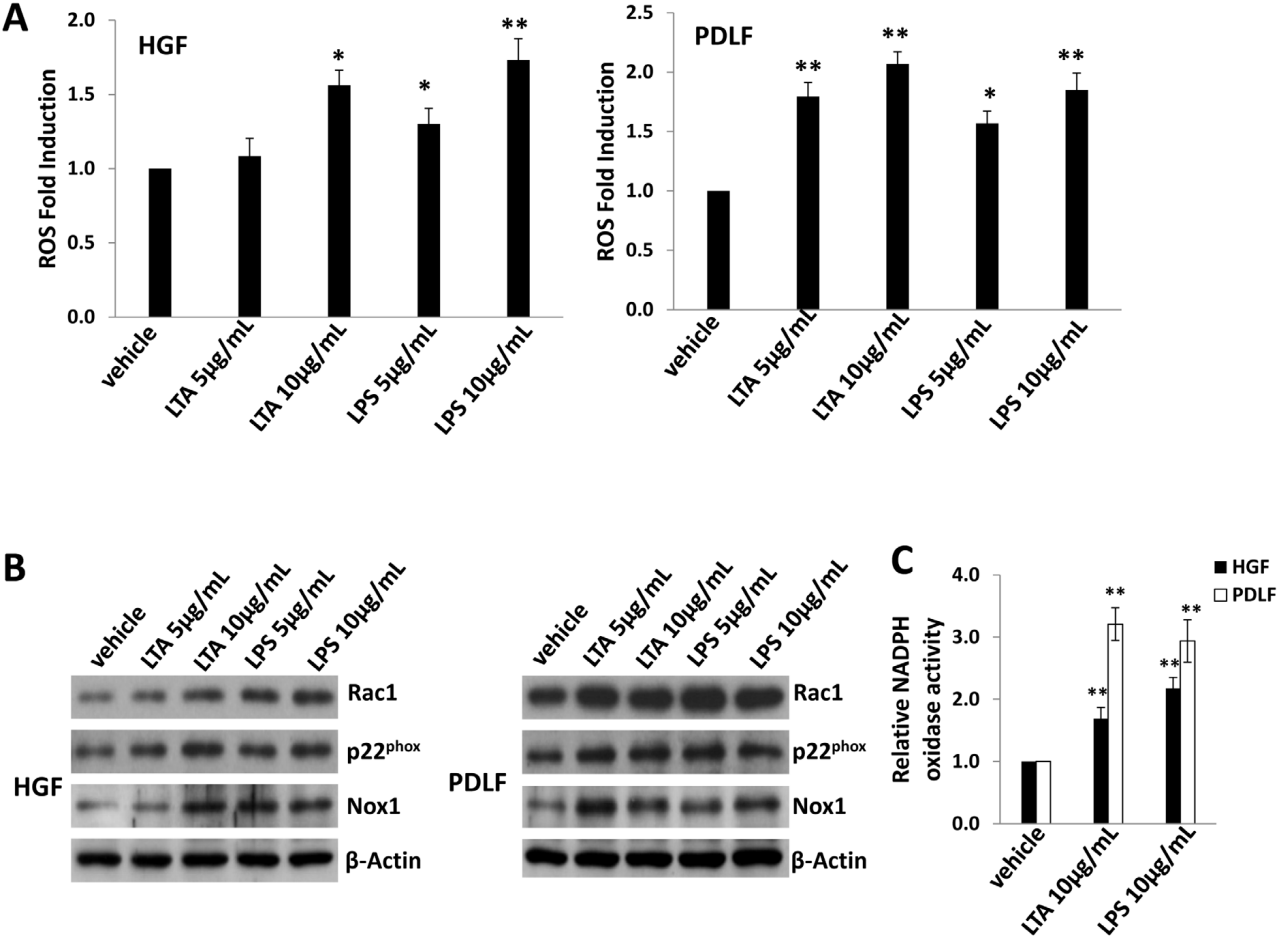
LTA and LPS from periodontal pathogenic bacteria induce ROS production from oral cells through increasing NADPH oxidase complex activities. (A) HGF and PDLF cells were treated with indicated concentrations of LTA from *S. aureus* or LPS from *P. gingivalis* for 24 h, then intracellular ROS production was measured as described in Methods. (B–C) Cells were treated as above, then proteins expression was detected by immunoblots (B), and NADPH oxidase activities were measured using a chemiluminescence-based assay as described in Methods (C). Error bars represent the standard errors of the means for 3 independent experiments. * = p<0.05, ** = p<0.01.

Besides the cellular receptors, we try to identify whether other host factors are involved in periodontal bacterial LTA and LPS facilitating KSHV infection as well. Recently, Bottero *et al* reported that ROS was induced by KSHV very early during primary infection of endothelial cells to promote virus entry [43], and they also found that pretreatment of the virus with heparin abolished ROS induction to block virus entry. Therefore, we seek to understand whether ROS, the co-factor for KSHV entry, is also related to bacterial LTA and LPS facilitating KSHV infection of oral cells. Using a ROS-specific dye, 5-(and-6)-chloromethyl-2′,7′-dichlorodihydrofluorescein diacetate, acetyl ester (CM-H2DCFDA) [43], we found that either *S. aureus*-LTA or *P. gingivalis-*LPS pretreatment induced significant intracellular ROS production from HGF and PDLF (Fig. 4A). As we know, ROS production requires the NADPH oxidase complex, which contains various NADPH oxidases (Nox1–4) and cytosolic components (NQO1, Rac1, p22^phox^, p47^phox^), depending on the stimulus signals and cell types [44], [45]. Here, we demonstrated that periodontal bacterial LTA and LPS predominantly increased the expression of Rac1/p22^phox^ /Nox1 cascade (Fig. 4B). We also tested other NADPH oxidase complex members including p47^phox^, Nox2 and Nox4, but no significant changes for these proteins were found in our study (Fig. S4). In functional validation, we confirmed that periodontal bacterial LTA and LPS significantly elevated the activities of NADPH oxidases within HGF and PDLF (Fig. 4C), using a luminescence-based biochemical assay as described previously [46].

**Figure 5:**
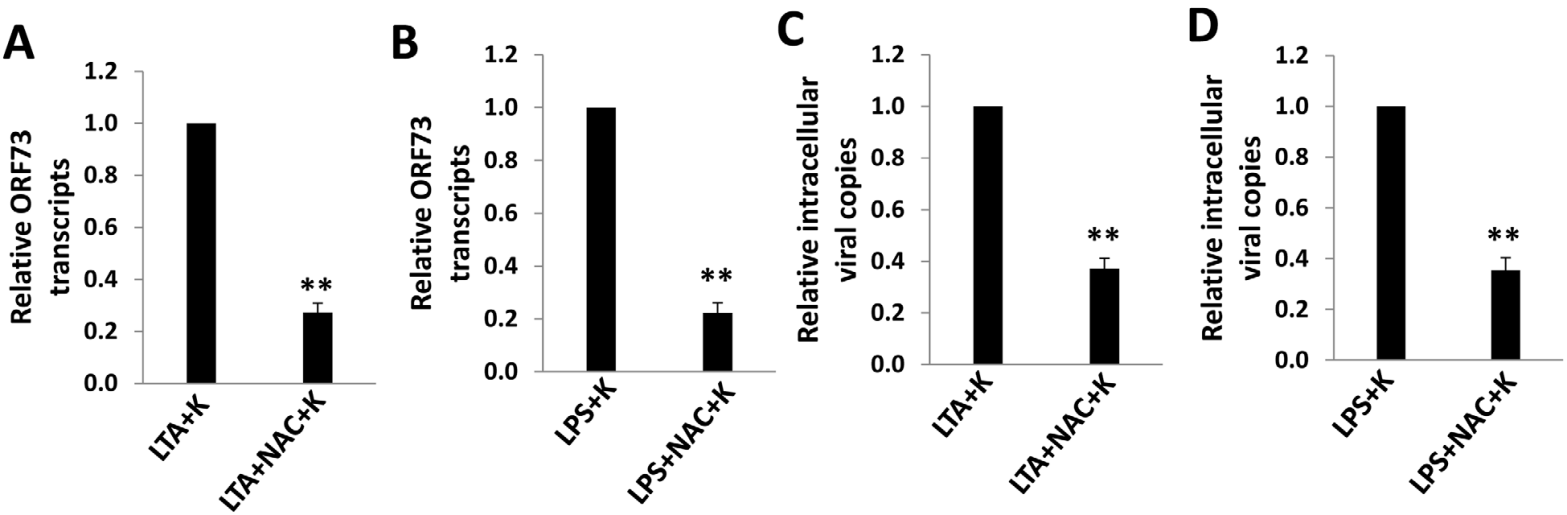
The antioxidant NAC reduces viral entry and gene expression during KSHV *de novo* infection. (A, C) HGF cells were pre-treated with 10 µg/mL of LTA from *S. aureus* or LPS from *P. gingivalis* for 24 h, then treated with or without NAC (10 mM) for 2 h, followed by infected with KSHV for 2 h and internalized viral DNA copies were measured by qPCR. (B, D) HGF were pretreated and infected as above, then treated with or without NAC (1 mM) for additional 24 h and *Lana* transcripts were measured by qRT-PCR. Error bars represent the standard errors of the means for 3 independent experiments. ** = p<0.01.

To confirm the role of ROS in KSHV infection to oral cells, we employed one of H_2_O_2_ scavengers, the antioxidant *N*-acetylcysteine (NAC), which had been reported to inhibit KSHV entry and consequently gene expression, as well as repress KSHV-related malignancies *in vivo* through blocking ROS production [47], [48]. We found that NAC treatment effectively inhibited both KSHV entry and subsequent latent gene expression from LTA- or LPS-pretreated HGF cells (Fig. 5). Similar results were obtained from LTA- or LPS-pretreated PDLF cells as well (Fig. S5).

### The MAPK and NF-κB signaling pathways are required for viral gene expression induced by periodontal bacterial LTA and LPS in oral cells

**Figure 6:**
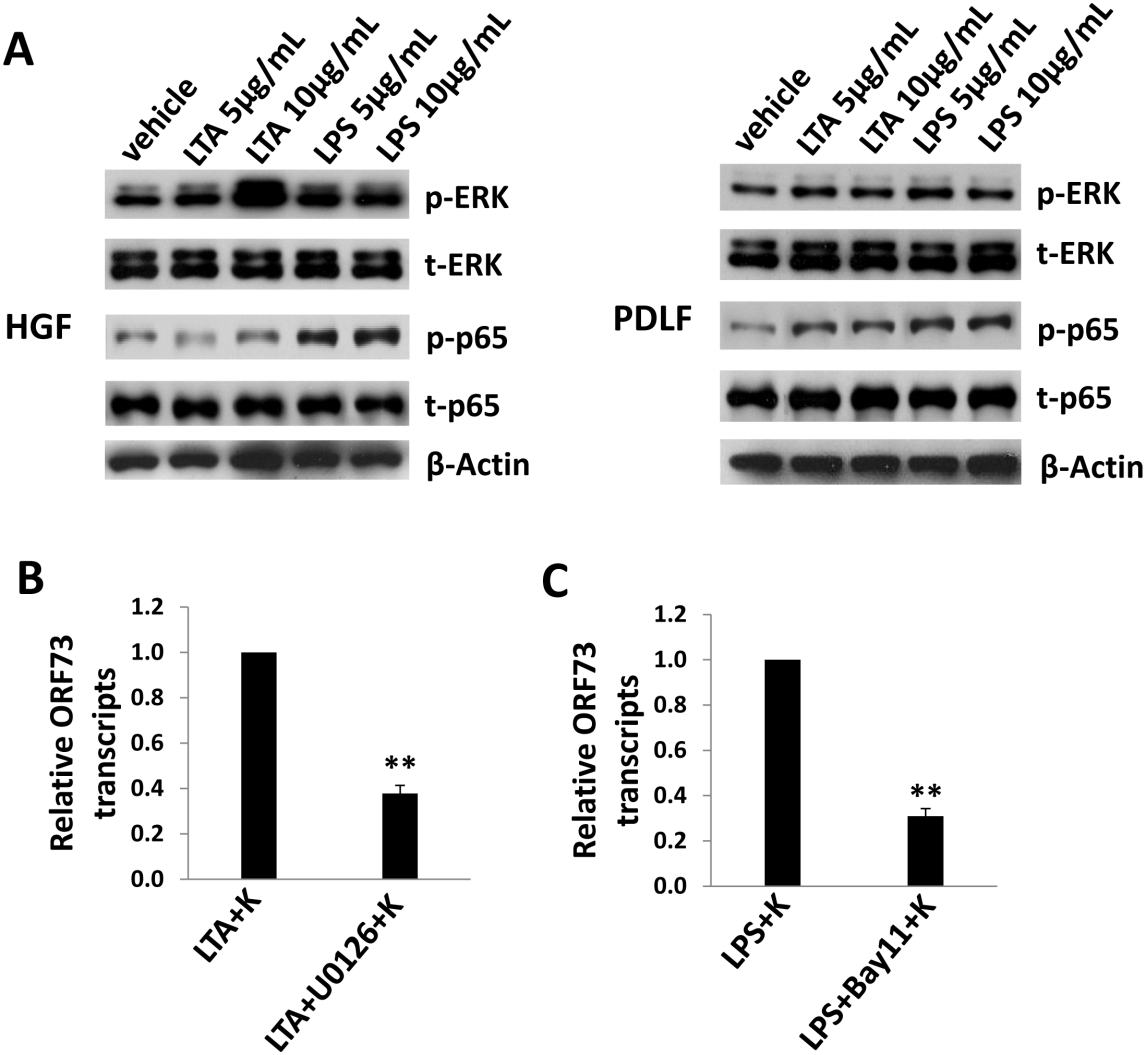
LTA and LPS from periodontal pathogenic bacteria increase viral latent gene expression through activating intracellular signaling pathways. (A) HGF and PDLF cells were pre-treated with indicated concentrations of LTA from *S. aureus* or LPS from *P. gingivalis* for 24 h, then infected with purified KSHV (MOI∼3). Proteins expression was detected by immunoblots. (B–C) HGF were pre-treated with 10 µg/mL of LTA from *S. aureus* or LPS from *P. gingivalis* for 24 h, then treated with 10 µM of the MEK/MAPK inhibitor U0126 or NF-κB inhibitor Bay11-7082 for 1.5 h, respectively, followed by infection with KSHV. qRT-PCR was used to quantify *ORF73* (*Lana*) transcripts. Error bars represent the standard errors of the means for 3 independent experiments. ** = p<0.01.

Once KSHV entry, the virus needs to interact with a variety of cellular molecules including some intracellular signaling pathways such as MAPK and NF-κB, for successful establishment of latent infection [49]–[52]. Therefore, we are interested to know whether these signaling pathways are involved in periodontal bacterial LTA- and LPS-promoting viral gene expression in oral cells. By using immuoblots, we found that LTA from *S. aureus* selectively induced MAPK-ERK phosphorylation and LPS from *P. gingivalis* increased NF-κB p65 phosphorylation in HGF cells, respectively (Fig. 6A). In contrast, in PDLF cells, both bacterial LTA and LPS apparently induced NF-κB p65 phosphorylation, while little impacts on MAPK-ERK activities (Fig. 6A). To confirm the role of MAPK and NF-κB pathways, we blocked these signaling pathways with their selective inhibitors, U0126 (for MAPK) and Bay11-7082 (for NF-κB), respectively. As shown in Fig. 6B–C, U0126 treatment significantly reduced viral gene expression from *S. aureus* LTA-treated HGF cells, while Bay11-7082 treatment greatly decreased viral gene expression from *P. gingivalis* LPS-treated HGF cells. Not surprisingly, neither U0126 nor Bay11-7082 treatment was able to affect viral entry within LTA- or LPS-pretreated cells (Fig. S6A–B), because the MAPK or NF-κB pathways play their roles at post-entry stages during KSHV *de novo* infection [49], [51], [52]. Moreover, immunoblots data confirmed that blocking the MAPK or NF-κB pathways was not able to alter HSPG expressional level in HGF cells (Fig. S6C).

## Discussion

**Figure 7:**
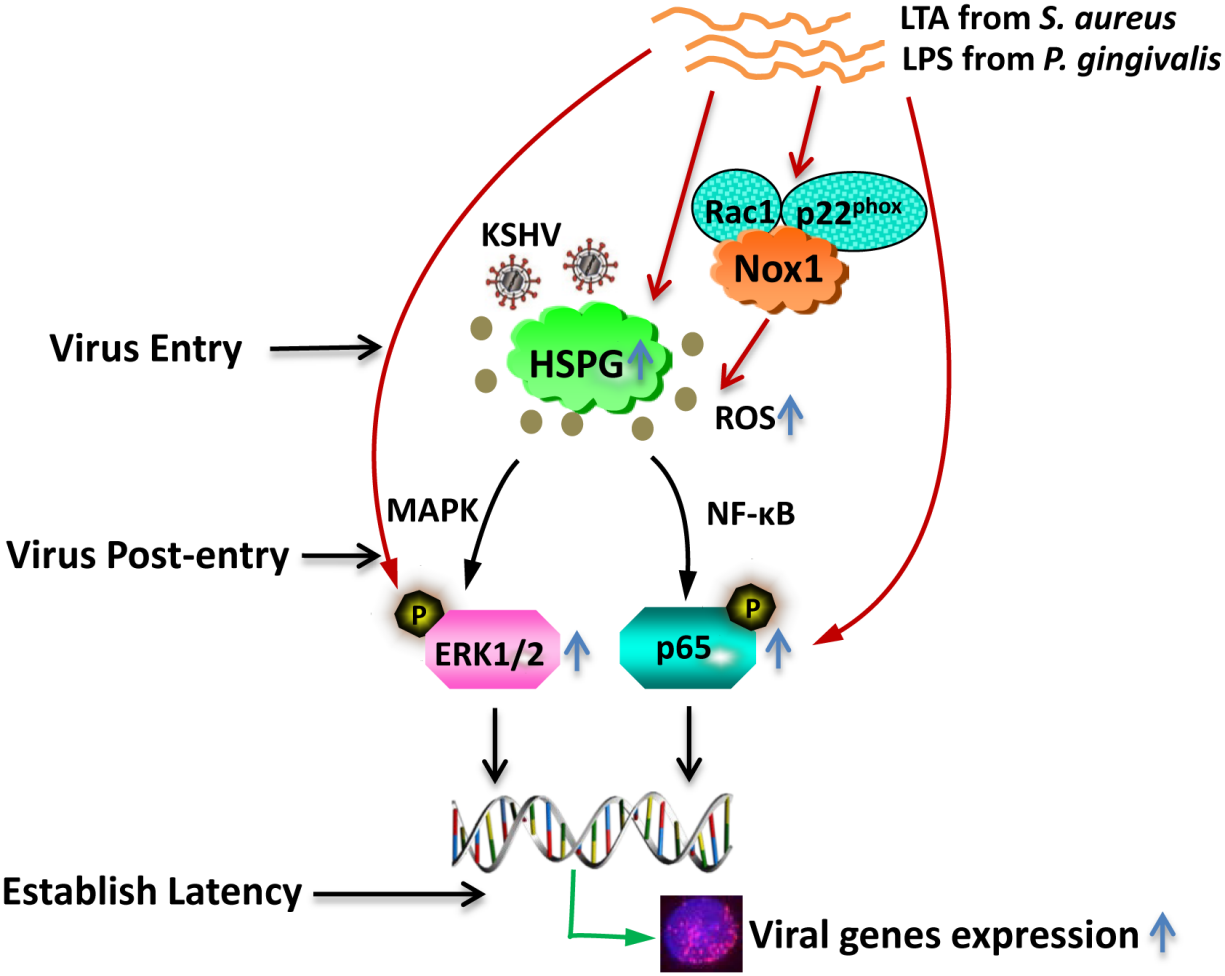
Schematic representation of mechanisms for facilitating KSHV entry and latency establishment in oral cells by periodontal bacterial LTA and LPS.

Oral cavity involvement represents the initial manifestation of KS in 20–60% of HIV-associated cases [53]–[55], and oral KS lesions contain higher KSHV viral loads relative to skin KS lesions [56]. As mentioned above, there is a significantly higher prevalence of severe oral inflammation and periodontal disease for HIV-positive patients [14], [15]. However, it remains unknown about the roles of oral pathogenic bacteria in KSHV infection and consequently KS development. To our knowledge, this is the first data demonstrating how periodontal bacterial productions facilitating oncogenic KSHV infection in oral cells via the complex mechanisms occurred at varied virus entry and post-entry stages (as summarized in Fig. 7). In fact, increasing KSHV infection by periodontal bacterial species makes oral cavity become a suitable reservoir place and promotes potential virus dissemination once stimulated into lytic reactivation. Interestingly, one very recent study has revealed that short-chain fatty acids produced by periodontal pathogens including *P. gingivalis* and *Fusobacterium nucleatum* induce KSHV lytic reactivation and promote virus replication [57]. In addition, our previous study has demonstrated that skin KS tumor tissues at more advanced stage contain a larger number of KSHV-infected cells (nodule >plaque >patch) [58], although such clinical relevance in oral KS tumors still requires further validation.

Another remaining question is how these periodontal bacterial LTA or LPS can stimulate HSPG, ROS and intracellular signaling pathways within oral cells found in the current study. PAMPs usually need to interact with respective PRRs for applying their biological functions in host cells, and one of the major PRRs is Toll-like receptors (TLRs) [59]. Bacterial LTA and LPS are well-known agonists to TLR2 and TLR4, respectively, which subsequently stimulate different downstream signaling pathways including MAPK or NF-κB [28], [29]. Interestingly, several TLRs have been found to be related with KSHV infection, replication and pathogenesis [60]–[63]. TLR3 is upregulated during KSHV infection of human monocytes and induces TLR3-specific cytokines and chemokines production [60]. Stimulation of TLR7/8 can reactivate latent KSHV and induce viral lytic gene transcription and replication [62]. In contrast, one recent study reports that KSHV-microRNAs can directly target IRAK1 and MYD88, two components of the TLR/IL-1R signaling cascade, to reduce inflammatory-cytokine expression [63]. Therefore, future study will try to identify whether and which TLRs and related molecules in cascade are used by periodontal bacterial LTA or LPS to facilitate KSHV infection of oral cells.

Our current study only focuses on LTA from *S. aureus* and LPS from *P. gingivalis*, their impacts on KSHV infection. Therefore, it is necessary to understand whether LTA and/or LPS from other oral pathogenic bacteria or even other bacterial PAMPs have similar roles for KSHV infection in oral cavity. Interestingly, one recent study demonstrates that *P. gingivalis* LPS and *Escherichia coli* LPS differently regulate cytokine production in human gingival fibroblasts [64]. Another recent study indicates that whole blood cell cultures (WBCC) populations obtained from healthy and chronic periodontitis patients may differ in the cytokine response to *P. gingivalis* LPS but not *E. coli* LPS [65]. In fact, *P. gingivalis-*derived LPS exhibits unique features compared with the LPS of other species, including differences in the structure of the O-antigen, as well as in the acylation patterns and receptor-activating capacities of the lipid A component [66]–[68]. Therefore, we assume that LTA and/or LPS from different bacterial species may have distinct impacts on host factors or immune response related to KSHV infection, although which requires further experimental validation. On the other hand, as periodontal pockets accommodate a multitude of bacterial phylotypes, sometimes it is difficult to differentiate between commensals and true pathogens to periodontal diseases or oral cancers development (especially in those immunocompromised patients) [69].

In the current study, we used a range of 2.5–10 µg/mL of *S. aureus*-derived LTA or *P. gingivalis*-derived LPS, that represents general concentrations used in other *in vitro* studies [70]–[73]. However, it is still lack of data describing the physiological concentrations of bacterial LTA and/or LPS in the oral cavity, especially HIV^+^ patients. To answer that question, we are planning to collect and compare the concentrations of total LTA and LPS within saliva samples from HIV^+^KSHV^+^ and HIV^+^KSHV^−^ patients, by using several commercial ELISA kits (such as Novatein Bioscience and Cloud-Clone). Notably, all these commercial ELISA kits can only be used for measure the total levels of LTA and/or LPS, but not for the levels of LTA and/or LPS derived from single species including *S. aureus* and *P. gingivalis*. Alternatively, we can measure the antibody titers specific for *S. aureus*-derived LTA or *P. gingivalis*-derived LPS within saliva using an ELISA-based method as described previously, which has demonstrated the significant elevated antibody titers for *P. gingivalis*-derived LPS in saliva from patients with periodontal diseases when comparing those from healthy controls [74].

Published data indicate the core oral microbiome consists of approximately 1000 species-level taxa [75], [76]. However, one recent study indicates the distinct and complex microbiome structures in human periodontitis and health revealed by 16S pyrosequencing [13]. The authors have found that community diversity is higher in disease, and 123 species are identified that are significantly more abundant in disease, and 53 in health. Based on this, we seek to determine whether KSHV co-infection will change the microbiome stucture in the oral cavity of HIV-positive patients, or different microbiome structures are present in HIV-positive patients with or without oral KS in future studies.

In summary, our data first time provide insights for complex mechanisms of periodontal bacteria-derived PAMPs especially LTA/LPS facillitating oncogenic herpesvirus infection and pathogenesis within primary oral cells. Our study will help to develop promising strategies targeting these bacteria-host-virus-associated mechanisms for reducing maintenance of KSHV in the oral cavity, and future clinical trials for treatment and/or prevention of oral KS in high-risk HIV-positive patients. For example, a readily available component of many commercial mouthwashes, chlorhexidine, has been found to reduce LTA concentrations within the oral cavity [77] and suppresses LTA-induced, TLR2-mediated inflammation [78].

## Materials and Methods

### Cell culture and reagents

KSHV-infected PEL cells (BCBL-1) were originally purchased from ATCC (kindly provided by Dr. Dean Kedes, University of Virginia) and maintained in RPMI 1640 media (Gibco) supplemented with 10% fetal bovine serum (FBS), 10 mM HEPES (pH 7.5), 100 U/mL penicillin, 100 µg/mL streptomycin, 2 mM L-glutamine, 0.05 mM β-mercaptoethanol, and 0.02% (wt/vol) sodium bicarbonate. Primary human gingival fibroblasts (HGF) and periodontal ligament fibroblasts (PDLF) were originally purchased from ScienCell by Dr. Amy Bradshaw (Medical University of South Carolina) and kindly provided to our laboratory. These cells were maintained in Dulbecco’s modified Eagle’s medium (DMEM, Mediatech) supplemented with 10% FBS, 10 mM HEPES (pH 7.5), 100 U/mL of penicillin, 100 µg/mL streptomycin, and 0.25 µg/mL amphotericin B. HeLa cells were maintained in Dulbecco’s modified Eagle’s medium (DMEM; Gibco) supplemented with 10% FBS, 100 U/ml of penicillin, and 100 µg/ml streptomycin. The LTA derived from *S. aureus* and LPS from *P. gingivalis* were purchased from InvivoGen, and the purities were more than 99.5% as described by the manufacturer.

### Virus purification and infection assays

To obtain KSHV for infection experiments, BCBL-1 cells were incubated with 0.6 mM valproic acid for 6 days. Following two low-velocity centrifugation steps to remove BCBL-1 cells, KSHV was purified from culture supernatants through ultracentrifugation at 20,000 g for 3 h, 4°C. Light microscopy was used subsequently to ensure that no intact BCBL-1 cells were retained during viral purification. The viral pellet was resuspended in 1/100 original volume in the appropriate culture media, and aliquots frozen at –80°C. Six-well plates containing HeLa cells were then incubated with serially diluted virus for 2 h, washed, and incubated in media for an additional 18–24 h. Immunofluorescence assays (IFA) to quantify expression of the KSHV latency-associated nuclear antigen (LANA) were then used to determine infectious viral titers by examining slides at 63X magnification using a Nikon TE2000-E fluorescence microscope. LANA exhibits punctate expression (“dots”) within the nucleus of infected cells using this IFA protocol, and the number of LANA dots correlates with viral episome copy number, as LANA tethers the viral episome to host cell chromosomes [79], [80]. Therefore, assuming that one LANA dot corresponds to a single viral episome in these assays, titers of our KSHV stocks approximated 4–5×10^6^ infectious particles/mL. HGF or PDLF were incubated with dilutions (MOI∼3) of viral stocks in the presence of 8 µg/mL Polybrene (Sigma) for 2 h at 37°C, and LANA IFA (see below) were used to quantify viral episomes within cells within 24 h of viral incubation with visualization at least 100 cells.

### Inhibition of signal transduction

The MEK/MAPK inhibitor U0126 and the NF-κB inhibitor Bay11-7082 were reconstituted according to the manufacturer’s instructions (Sigma). U0126 and Bay11-7082 were added to cell cultures for 1.5 h, then changed with fresh medium for additional 24 h-incubation, and perturbations in signal transduction were confirmed using immunoblot assays (see below).

### Immunoblotting

Cells were lysed in buffer containing 20 mM Tris (pH 7.5), 150 mM NaCl, 1% NP40, 1 mM EDTA, 5 mM NaF and 5 mM Na_3_VO_4_. Total cell lysates (30 µg) were resolved by 10% SDS–PAGE, transferred to nitrocellulose membranes, and immunoblotted using 100–200 µg/mL antibodies, including p-ERK/t-ERK, NF-κB p-p65/t-p65, Rac1 (cell signaling), Nox1, HSPG (Abcam), p22^phox^, p47^phox^, Nox2, Nox4 (Santa Cruz). For loading controls, blots were reacted with antibodies detecting β-Actin (Sigma). Immunoreactive bands were developed using an enhanced chemiluminescence reaction (Perkin-Elmer) and visualized by autoradiography.

### Immunofluorescence Assays (IFA)

Briefly, 1×10^4^ HGF or PDLF per well were seeded in eight-well chamber slides (Nunc) and incubated with serial dilutions of viral stocks in the presence of 8 µg/mL Polybrene (Sigma) for 2 h at 37°C. After remaining in culture for 24 h, cells were incubated in 1∶1 methanol-acetone at 20°C for fixation and permeabilization, then with a blocking reagent (10% normal goat serum, 3% bovine serum albumin, and 1% glycine) for an additional 30 min. Cells were then incubated for 1 h at 25°C with 1∶1000 dilution of a rat monoclonal antibody (ABI) recognizing LANA of KSHV or 1∶400 dilution of a rat monoclonal antibody for HSPG (Abcam), followed by 1∶200 dilution of a goat anti-rat secondary antibody conjugated to Texas Red (Invitrogen). For identification of nuclei, cells were subsequently counterstained with 0.5 µg/mL 4′,6-diamidino-2-phenylindole (DAPI; Sigma) in 180 mM Tris-HCl (pH 7.5), washed and prepared for visualization using a Nikon TE2000-E fluorescence microscope.

### Virus Entry Blocking Assays

Cells were first treated with 0.4 mg/mL mannan (Sigma) or 20 µg/mL xCT Ab (Santa Cruz), or purified virions were first treated with 0.5 mg/mL heparin (Sigma) or 15 µg/mL soluble integrin α3β1 and αvβ3 (Upstate Biotechnology) for 1 h at 4°C, then these cells were infected with purified virions (MOI∼3) for 2 h at 37°C. After that, cells were trypsinized and washed to remove extracellular KSHV virions. The internalized KSHV were measured by Real-time qPCR as described below.

### ROS measurement

Oral cells cultured in a 12-well plate were loaded with 10 µM of the ROS dye c-H2DCFDA (Invitrogen) for 1 h at 37°C in Hanks’ Balanced Salt Solution (HBSS) containing Ca and Mg. Cells were then wash once with HBSS/Ca/Mg once to remove dye, resuspended in growth medium for 1 h. Fluorescence was measured using a Synergy HT microplate reader (BioTek Instruments) with a 485/20 excitation, 528/20 emission filter pair and a photomultiplier tube (PMT) sensitivity setting of 55. For NAC treatment assays, cells were either mock treated or pretreated with NAC (10 mM) for 2 h, after which they were infected with KSHV (MOI∼3) for 2 h and internalized viral DNA copies were measured by qPCR as described below. For some cells after the virus was removed, they were mock treated or treated with NAC (1 mM) for additional 24 h, then LANA expression was measured by qRT-PCR as described below.

### PCR

Total cellular DNA was prepared using the QIAamp DNA Mini-kit according to the manufacturer’s instructions (Qiagen). Briefly, cells were trypsinized for 5 min at 37°C and were collected with 1 mL of ice-cold DMEM. Cells were pelleted at 2,000 rpm for 5 min, washed, and resuspended in 200 µL of 1xPBS, and total DNA was prepared according to the manufacturer’s instructions. To ensure that viral DNA amplification in these experiments was not the result of “carryover” viral DNA from culture supernatants rather than intracellular virus, cells were washed several times in fresh medium prior to trypsinization, and samples from culture supernatants following these washes were assessed for viral DNA content. For qRT-PCR experiments, total RNA was isolated using the RNeasy Mini kit according to the manufacturer’s instructions (Qiagen). cDNA was synthesized from equal total RNA using SuperScript III First-Strand Synthesis SuperMix Kit (Invitrogen) according to the manufacturer’s procedures. Primers designed for amplification of target genes are listed as below: *Lana sense 5′ TCCCTCTACACTAAACCCAATA 3′; Lana antisense 5′ TTGCTAATCTCGTTGTCCC 3′; β-actin sense 5′ GGAAATCGTGCGTGACATT 3′; β-actin antisense 5′ GACTCGTCATACTCCTGCTTG 3′*. Amplification experiments were carried out using an iCycler IQ Real-Time PCR Detection System, and cycle threshold (Ct) values were tabulated in duplicate for each gene of interest for each experiment. “No template” (water) controls were used to ensure minimal background contamination. Using mean Ct values tabulated for different experiments, and using Ct values for β-actin as loading controls, fold changes for experimental groups relative to assigned controls were calculated using automated iQ5 2.0 software (Bio-rad).

### Cell viability assays

Cell viability was assessed using a standard MTT assay as previously described [58]. A total of 5×10^3^ HGF or PDLF cells were incubated in individual wells in a 96-well plate for 24 h. Indicated concentrations of LTA or LPS were added and after 24 h, cells were incubated in 1 mg/mL of MTT solution (Sigma-Aldrich) at 37°C for 3 h then 50% DMSO overnight and optical density at 570 nm determined by a spectrophotometer (Thermo Labsystems).

### Flow cytometry

Following trypsinization, HGF and PDLF cells were resuspended in staining buffer (3% BSA in 1×PBS) for 20 minutes, then incubated on ice for 30 min with 1∶50 dilution of primary antibodies α3β1 and αvβ3 (Millipore), xCT (Santa Cruz), DC-SIGN (R&D). Following two subsequent wash steps, cells were incubated for an additional 30 min with 1∶200 dilution of secondary antibodies (Invitrogen) including goat anti-mouse IgG Alexa Fluor 647 (for detecting α3β1, αvβ3 and DC-SIGN) or Donkey anti-goat IgG Alexa Fluor 647 (for detecting xCT). Controls included cells incubated with secondary antibodies only. Cells were resuspended in 1× PBS and analyzed using a FACS Calibur 4-color flow cytometer (BD) and FlowJo software (TreeStar) to quantify cell surface localization of target proteins.

### NADPH oxidase activities assays

The chemiluminescence-based NADPH oxidase activities assays were performed as described previously with modifications [46]. Briefly, cells were gently scraped and centrifuged at 500 g for 10 min at 4°C. The cell pellet was resuspended with 35 µL ice-cold lysis buffer and kept on ice for 20 min. To a final 200 µL of HBSS/Ca/Mg buffer containing NADPH (1 µM, Sigma) and lucigenin (20 µM, Sigma), 5 µL of cell lysates was added to initiate the reaction for 5 min at 37°C. Chemiluminescence was measured immediately using a Synergy HT microplate reader (BioTek Instruments).

### Statistical analyses

Significance for differences between experimental and control groups was determined using the two-tailed Student’s t-test (Excel 8.0).

## Supporting Information

Figure S1 **LTA and LPS from periodontal pathogenic bacteria do not affect oral fibroblast viability.** HGF and PDLF were treated with indicated concentrations of LTA from *S. aureus* or LPS from *P. gingivalis* for 24 h, respectively. Cell viability was assessed by the standard MTT assays as described in Methods. Error bars represent the standard errors of the means for 3 independent experiments.
(TIF)

Figure S2
**Heparin treatment blocks KSHV entry into HGF cells.** (A–B) HGF were incubated with 10 µg/mL LTA or LPS for 24 h, and purified virions (MOI∼3) were incubated with or without 0.5 mg/mL heparin for 1 h at 4°C. Cells were subsequently infected for 2 h at 37°C, then DNA (2 h p.i.) and RNA (24 h p.i.) were isolated for quantification of intracellular viral copies or *ORF73* (*Lana*) transcripts using qPCR (A) or qRT-PCR (B), respectively. Error bars represent the standard errors of the means for 3 independent experiments. **/##/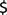 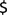 = p<0.01 relative to K (**), LTA+K (##), and LPS+K (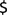 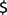).(TIF)

Figure S3 **Cellular receptors for KSHV entry on oral fibroblasts influenced by LTA and LPS from periodontal pathogenic bacteria.** HGF and PDLF were treated with or without 10 µg/mL of LTA from *S. aureus* or LPS from *P. gingivalis* for 24 h, then expression of cellular receptors for KSHV entry including Integrin α3β1, αvβ3, xCT and DC-SIGN, on cell-surface were detected by flow cytometry as described in Methods.
(TIF)

Figure S4 **Nox2, Nox4 and p47** ^phox^ **are not affected by bacterial LTA and LPS.** HGF and PDLF cells were treated with indicated concentrations of LTA from *S. aureus* or LPS from *P. gingivalis* for 24 h, then proteins expression was detected by immunoblots.
(TIF)

Figure S5 **Blocking ROS production by the antioxidant NAC reduces viral entry and gene expression within PDLF cells.** (A, C) PDLF cells were pre-treated with 10 µg/mL of LTA from *S. aureus* or LPS from *P. gingivalis* for 24 h, then treated with or without NAC (10 mM) for 2 h, followed by infected with KSHV for 2 h and internalized viral DNA copies were measured by qPCR. (B, D) PDLF were pretreated and infected as above, then treated with or without NAC (1 mM) for additional 24 h and *Lana* transcripts were measured by qRT-PCR. Error bars represent the standard errors of the means for 3 independent experiments. ** = p<0.01.
(TIF)

Figure S6 **Blocking intracellular signaling activities is not able to affect KSHV entry into oral cells increased by LTA and LPS.** (A–B) HGF were pre-treated with 10 µg/mL of LTA from *S. aureus* (A) or LPS from *P. gingivalis* (B) for 24 h, then treated with 10 µM of the MEK/MAPK inhibitor U0126 (A) or NF-κB inhibitor Bay11-7082 (B) for 1.5 h, respectively, followed by incubation with KSHV for 2 h. qPCR was used to quantify internalized viral copies. Error bars represent the standard errors of the means for 3 independent experiments. (C) Proteins expression was detected by immunoblots.
(TIF)
