# Supplementary figures and images for: Lipoteichoic Acid (LTA) and Lipopolysaccharides (LPS) from Periodontal Pathogenic Bacteria Facilitate Oncogenic Herpesvirus Infection within Primary Oral Cells
Source: PLoS One. 2014 Jun 27;9(6):e101326. doi: 10.1371/journal.pone.0101326 (PMC4074159; doi:10.1371/journal.pone.0101326)

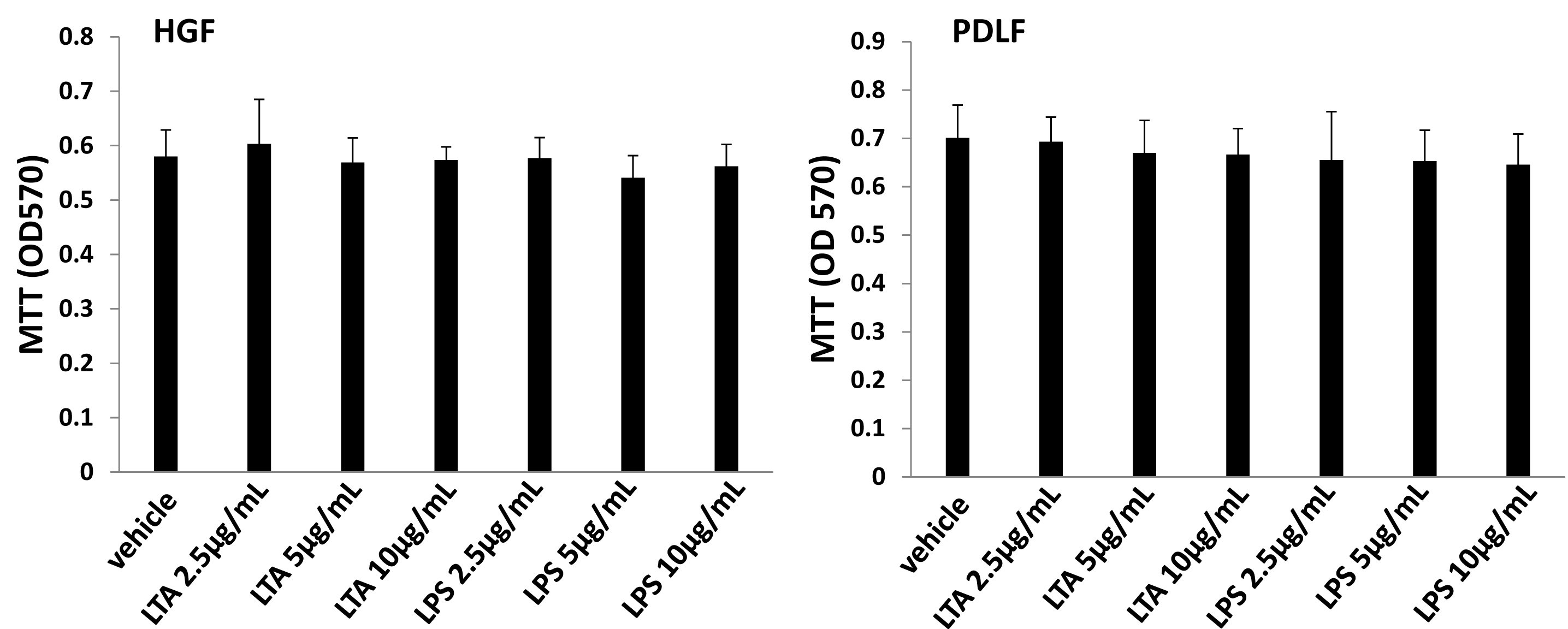

Supplement: Figure S1 — LTA and LPS from periodontal pathogenic bacteria do not affect oral fibroblast viability. HGF and PDLF were treated with indicated concentrations of LTA from S. aureus or LPS from P. gingivalis for 24 h, respectively. Cell viability was assessed by the standard MTT assays as described in Methods. Error bars represent the standard errors of the means for 3 independent experiments. (TIF) [file pone.0101326.s001.tif]

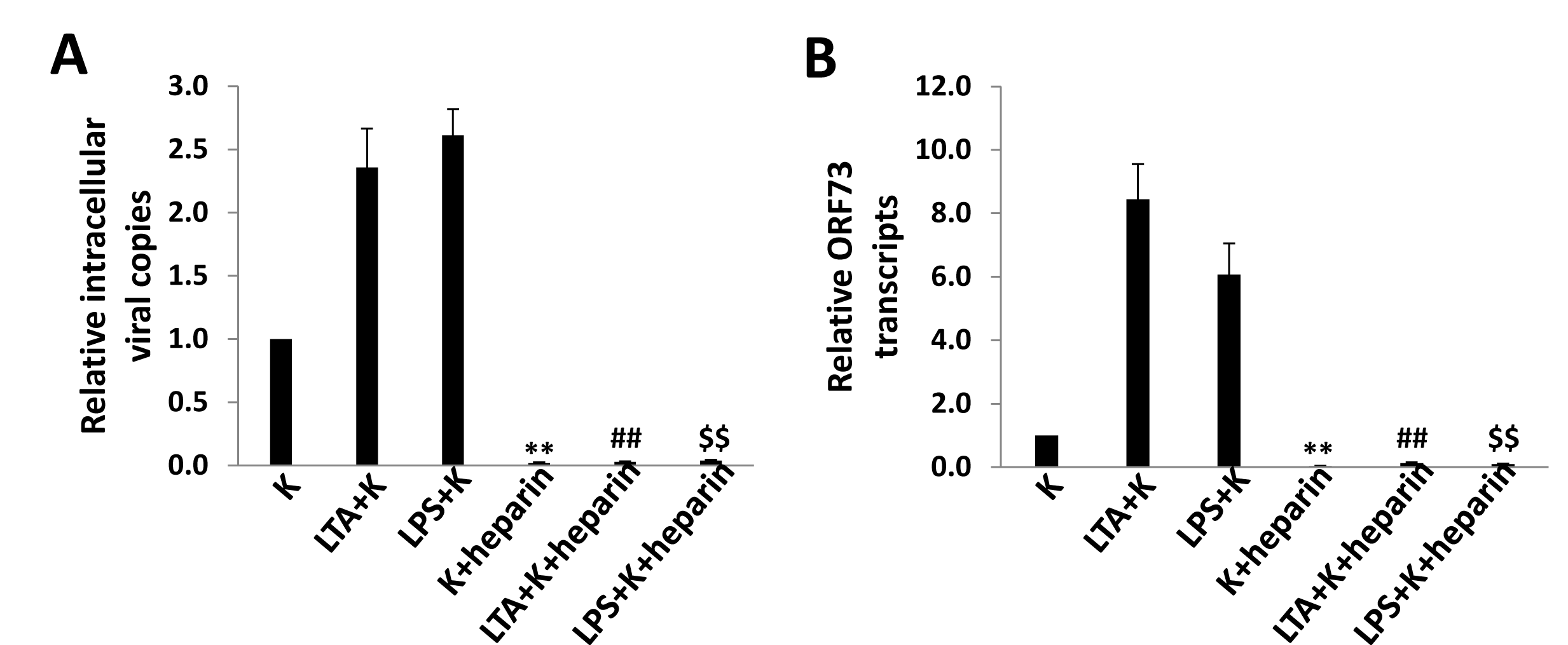

Supplement: Figure S2 — Heparin treatment blocks KSHV entry into HGF cells. (A–B) HGF were incubated with 10 µg/mL LTA or LPS for 24 h, and purified virions (MOI∼3) were incubated with or without 0.5 mg/mL heparin for 1 h at 4°C. Cells were subsequently infected for 2 h at 37°C, then DNA (2 h p.i.) and RNA (24 h p.i.) were isolated for quantification of intracellular viral copies or ORF73 (Lana) transcripts using qPCR (A) or qRT-PCR (B), respectively. Error bars represent the standard errors of the means for 3 independent experiments. **/##/ = p<0.01 relative to K (**), LTA+K (##), and LPS+K ( ). (TIF) [file pone.0101326.s002.tif]

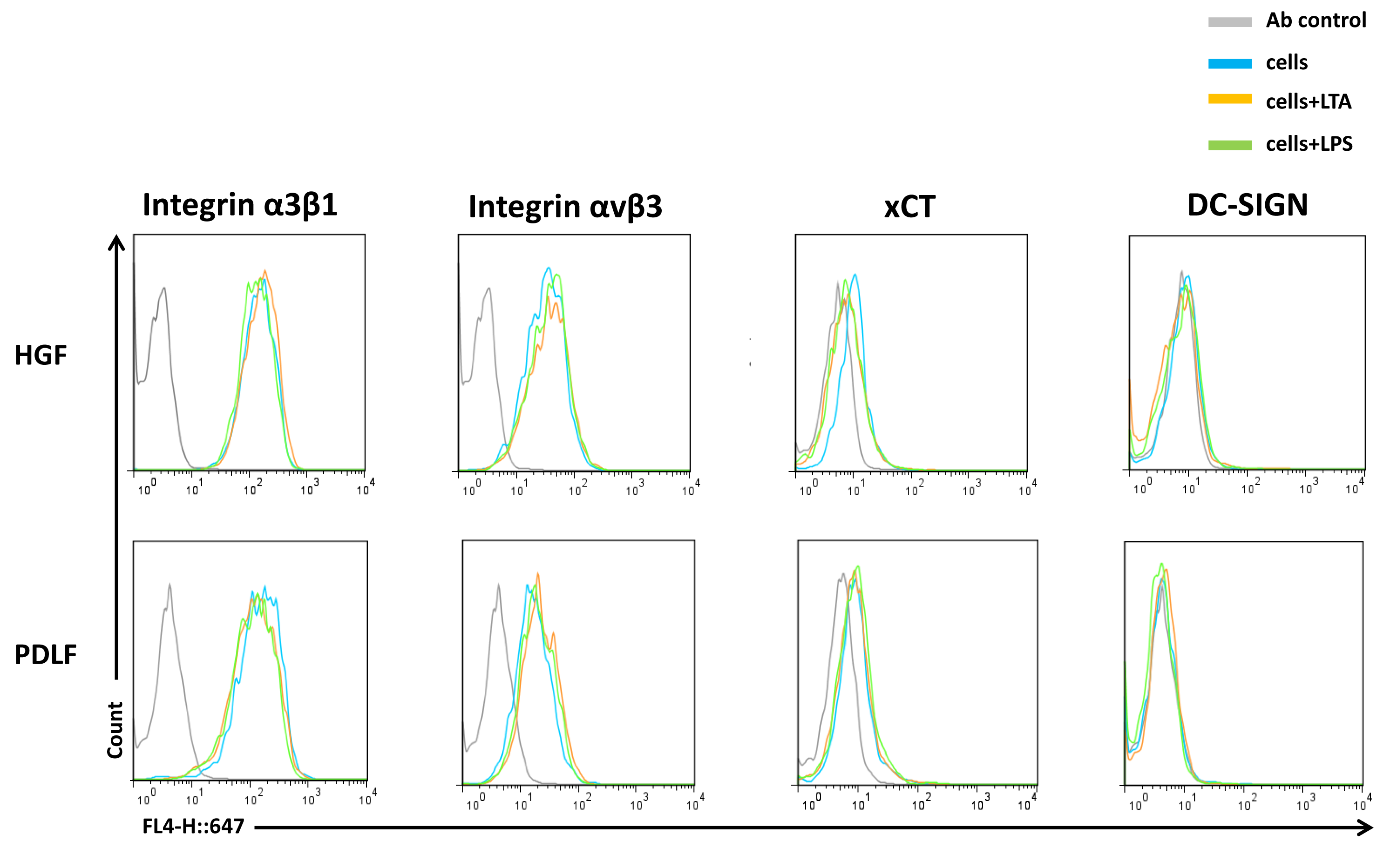

Supplement: Figure S3 — Cellular receptors for KSHV entry on oral fibroblasts influenced by LTA and LPS from periodontal pathogenic bacteria. HGF and PDLF were treated with or without 10 µg/mL of LTA from S. aureus or LPS from P. gingivalis for 24 h, then expression of cellular receptors for KSHV entry including Integrin α3β1, αvβ3, xCT and DC-SIGN, on cell-surface were detected by flow cytometry as described in Methods. (TIF) [file pone.0101326.s003.tif]

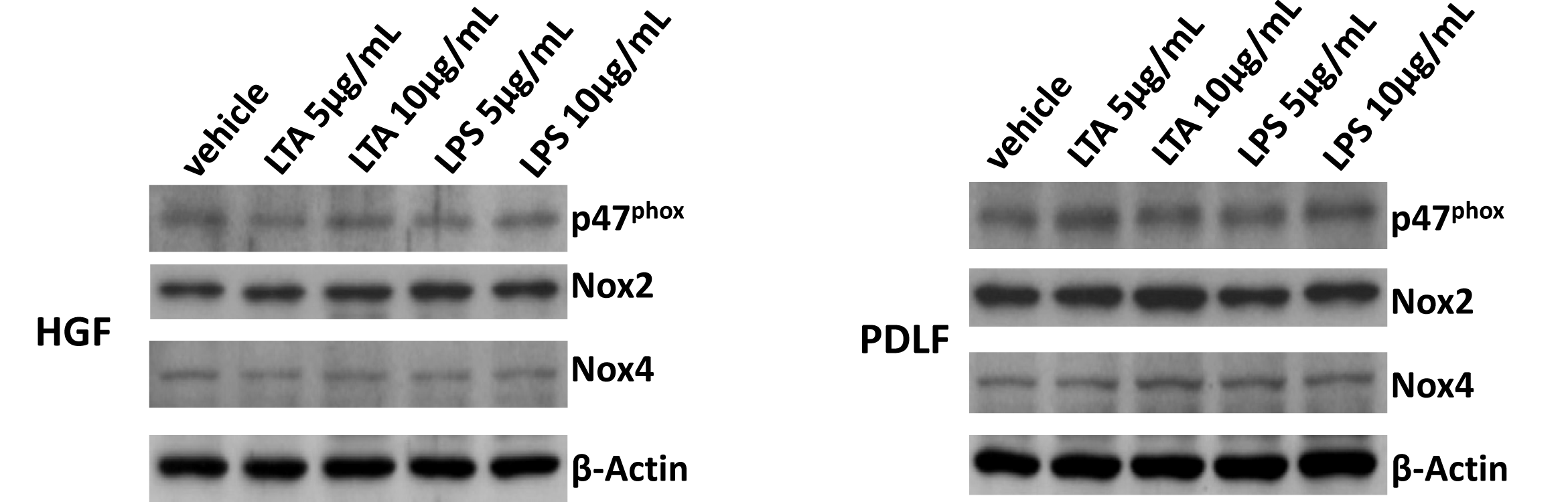

Supplement: Figure S4 — Nox2, Nox4 and p47 phox are not affected by bacterial LTA and LPS. HGF and PDLF cells were treated with indicated concentrations of LTA from S. aureus or LPS from P. gingivalis for 24 h, then proteins expression was detected by immunoblots. (TIF) [file pone.0101326.s004.tif]

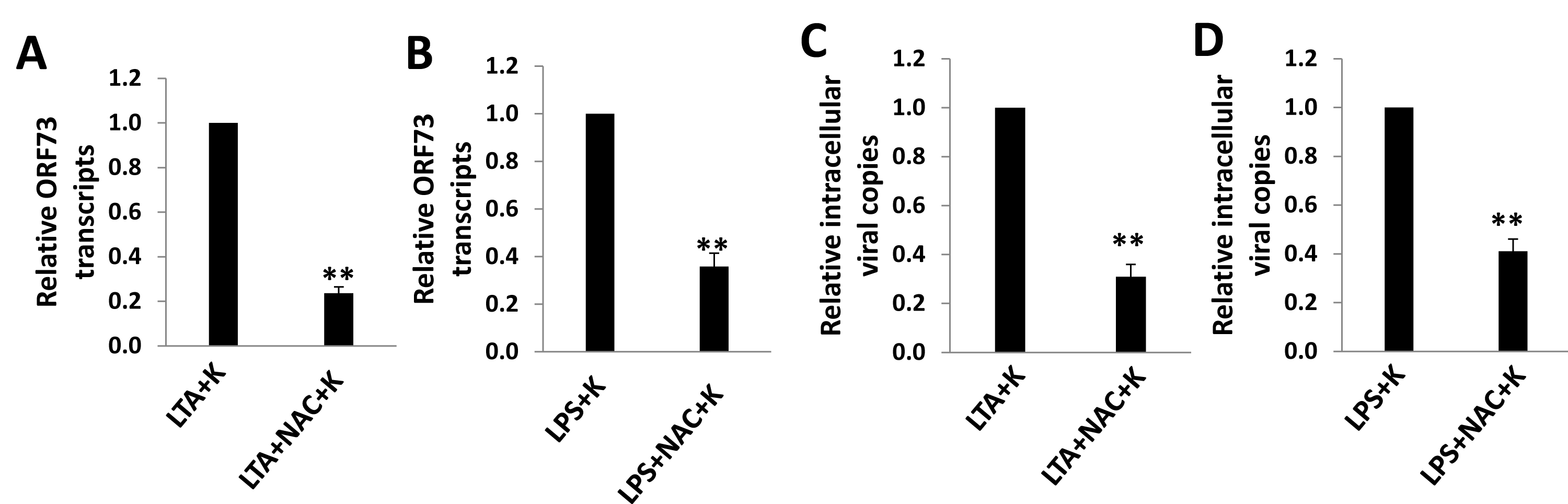

Supplement: Figure S5 — Blocking ROS production by the antioxidant NAC reduces viral entry and gene expression within PDLF cells. (A, C) PDLF cells were pre-treated with 10 µg/mL of LTA from S. aureus or LPS from P. gingivalis for 24 h, then treated with or without NAC (10 mM) for 2 h, followed by infected with KSHV for 2 h and internalized viral DNA copies were measured by qPCR. (B, D) PDLF were pretreated and infected as above, then treated with or without NAC (1 mM) for additional 24 h and Lana transcripts were measured by qRT-PCR. Error bars represent the standard errors of the means for 3 independent experiments. ** = p<0.01. (TIF) [file pone.0101326.s005.tif]

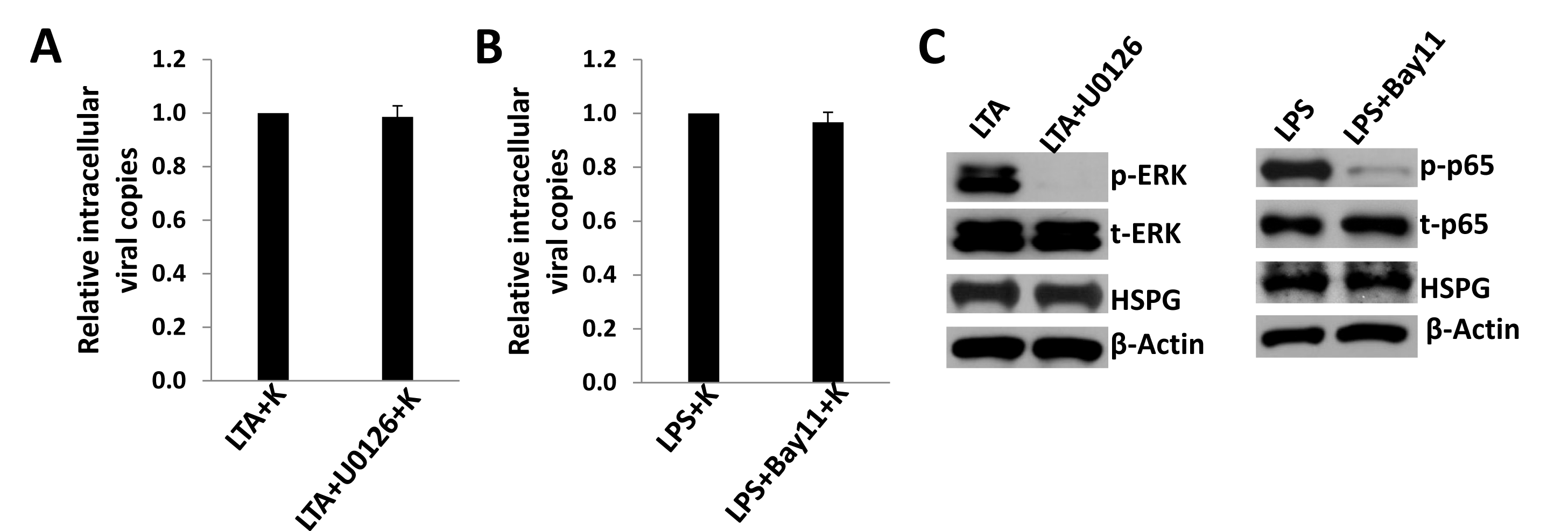

Supplement: Figure S6 — Blocking intracellular signaling activities is not able to affect KSHV entry into oral cells increased by LTA and LPS. (A–B) HGF were pre-treated with 10 µg/mL of LTA from S. aureus (A) or LPS from P. gingivalis (B) for 24 h, then treated with 10 µM of the MEK/MAPK inhibitor U0126 (A) or NF-κB inhibitor Bay11-7082 (B) for 1.5 h, respectively, followed by incubation with KSHV for 2 h. qPCR was used to quantify internalized viral copies. Error bars represent the standard errors of the means for 3 independent experiments. (C) Proteins expression was detected by immunoblots. (TIF) [file pone.0101326.s006.tif]
